# Factors influencing patient satisfaction with dental appearance and treatments they desire to improve aesthetics

**DOI:** 10.1186/1472-6831-11-6

**Published:** 2011-02-23

**Authors:** Mon Mon Tin-Oo, Norkhafizah Saddki, Nurhidayati Hassan

**Affiliations:** 1 School of Dental Sciences, Universiti Sains Malaysia, Health Campus, 16150 Kubang Kerian, Kelantan, Malaysia

## Abstract

**Background:**

We assessed factors influencing patients' satisfaction with their dental appearance and the treatments they desired to improve dental aesthetics.

**Methods:**

A cross-sectional study was performed out among 235 adult patients who visited the Hospital Universiti Sains Malaysia dental clinic. A structured, interviewer-guided questionnaire was used to identify patient satisfaction with their general dental appearance, cosmetic elements and desired treatments.

**Results:**

The 235 patients consisted of 70 males (29.8%) and 165 females (70.2%), of mean age 31.5 years (SD 13.0). Of these patients, 124 (52.8%) were not satisfied with their general dental appearance. In addition, 132 patients (56.2%) were not happy with the color of their teeth, 76 (32.3%), regarded their teeth were poorly aligned, 62 (26.4%), as crowded and 56 (23.4%) protruded. Dissatisfaction with tooth color was significantly higher in female than in male patients (odds ratio [OR] of 1.99 (95% confidence interval [CI] 1.13-3.50). Tooth whitening was the treatment most desired by patients (48.1%). Results of multiple logistic regression analysis showed that patient dissatisfaction with general dental appearance was significantly associated with female gender (OR = 2.18; 95% CI: 1.18-4.03), unhappiness with tooth color (OR = 3.05; 95% CI: 1.74-5.34) and the opinion that their teeth protruded (OR = 2.91, 95% CI: 1.44-5.91).

**Conclusions:**

Most patients in this study were not satisfied with their dental appearance with a greater percentage of females expressing dissatisfaction than males. An age was not associated with satisfaction. Unhappiness with tooth color and feelings of having protruding teeth also had a significant negative influence on patient satisfaction with general dental appearance.

## Background

Dental appearance is an important feature in determining the attractiveness of a face, and thus plays a key role in human social interactions. Among the significant factors affecting overall dental appearance are tooth color, shape, and position; quality of restoration; and the general arrangement of the dentition, especially of the anterior teeth [1]. Furthermore, an aesthetically pleasing smile was found to depend on tooth color, size, shape, and position, upper lip position, visibility of teeth and amount of gingival display [2]. Although each factor may be considered individually, all components must act together to create a harmonic and symmetric entity that produces the final aesthetic effect [1].

In general, people desire for pearly white teeth. Thus, tooth color is one of the most important factors determining satisfaction with dental appearance [1,3]. Self-satisfaction with tooth color decreases with increasing severity of discoloration [4,5]. White teeth have been positively correlated with high ratings of social competence, intellectual ability, psychological adjustment and relationship status [6]. Alternatively, untreated dental caries, non-aesthetic or discolored anterior teeth restorations and missing anterior teeth usually lead to dissatisfaction with dental appearance [3,7-9]. Furthermore, treatments improving dental aesthetics have been found to increase patient quality of life and psychological status [10,11].

Malocclusion is a common oral disorder, although treatment needs and demands vary. In some populations, tooth misalignments are not regarded as serious enough to necessitate treatment [12-14], whereas, in other populations, the need for orthodontic treatment may be very high [15,16]. There is general agreement that people are motivated to seek orthodontic treatment because of the negative physical, psychological and social impacts of malocclusion, but studies of the effects of malocclusion and its treatment on people's lives have yielded inconsistent results [17,18]. These discrepancies may be due to various interpretations of physical, psychological and social impact and lack of standardized methods to measure these quality of life constructs [17].

Currently, cosmetic dentistry has become an important aspect of dentistry. Tooth whitening treatments, anterior teeth restoration, labial veneers crowns, and orthodontic treatment are frequently demanded by patients who interested in improving their dental appearance [3]. We have assessed satisfaction with dental appearance, desired treatments to improve dental appearance, and factors that influence satisfaction with dental appearance among adult patients who attended the dental clinic at the Hospital Universiti Sains Malaysia (HUSM).

## Methods

This cross sectional study was carried out from June 1, 2009 to January 31, 2010 among patients who attended the HUSM dental clinic. All included patients were newly registered adults >18 years old, who had not received any dental treatment within the previous six months, were able to understand the Malay language and have no clear evidence of cognitive disturbances. Sample size was calculated using the formula estimating a single proportion with a requirement for 95% confidence [19]. The prevalence of dissatisfaction with dental appearance was estimated to be 62.7% based on the satisfaction with dental aesthetics among adult patients attending a military dental clinic in Tel Aviv, Israel [3]. Considering the available resources, a sample size of 183 was selected with a precision of 0.07 (7%). To accommodate for a 30% non-response rate, 238 patients were invited to participate in this study.

A systematic random sampling technique was used to select the study sample. The sampling interval was decided based on the estimated number of eligible patients attending the clinic on a normal outpatient day, with every tenth patient was invited to participate. No possible biases regarding the selection of the study population were anticipated and the samples were representative of the reference population. This study was approved by the Research and Ethics Committee (Human), Universiti Sains Malaysia.

A structured, interviewer guided questionnaire (Table 1) was used for data collection. The questionnaire consisted of questions on socio-demographic items including sex, age, and level of education, as well as questions on each patient's satisfaction with his/her then-current general dental appearance. Patients were also asked about their satisfaction with tooth color, perceived malalignment of teeth (crowding, poorly aligned or protruding), caries in anterior teeth, non-aesthetic anterior tooth color restoration and presence of tooth fracture. In addition, patients were asked to select the aesthetic treatments they wished to undergo, including orthodontic treatment, crowns, tooth whitening, tooth color restorations and partial dentures.

**Table 1:** Questionnaire used in the study

Survey on patient satisfaction with dental appearance and desired treatment to improve aesthetics			
1. Sex □Male □Female		2. Age:___________(years)	
3. Education level: □ Primary □ Secondary □ Post secondary □ Tertiary			
1.	Are you satisfied with the general appearance of your teeth?	□ Yes	□ No
2.	Are you satisfied with your tooth color?	□ Yes	□ No
3.	Do you feel your teeth are crowded?	□ Yes	□ No
4.	Do you feel your teeth are poorly aligned?	□ Yes	□ No
5.	Do you feel your teeth are protruding?	□Yes	□ No
6.	Do you have dental caries in your front teeth?	□ Yes	□ No
7.	Do you have non-aesthetic fillings in your front teeth?	□ Yes	□ No
8.	Do you have fractures in your front teeth?	□ Yes	□ No
9.	Do you wish to undergo these treatments to improve the appearance of your teeth?		
	a. Orthodontic treatment to realign teeth	□ Yes	□ No
	b. Tooth whitening	□ Yes	□ No
	c. Dental crowns	□ Yes	□ No
	d. Tooth coloured fillings	□ Yes	□ No
	e. Dentures	□ Yes	□ No

Prior to this study the clarity of the questionnaire was pre-tested on 15 patients who were not involved in the study. Feedback regarding problems understanding and answering the questionnaire was obtained and addressed. Each patient provided written informed consent before participation in this study.

Data were entered and analyzed using the Statistical Package for Social Sciences (SPSS) for Windows software (version 12.0; SPSS Inc, Chicago). Descriptive statistics such as mean and standard deviation (SD) for continuous variables and frequency and percentage for categorical variables were determined. The chi-square test was used to compare the sex, age, education levels of patients who were and were not satisfied with their dental appearance. The level of significance was set at 0.05.

Factors influencing patient satisfaction with dental appearance were determined at both the univariate and multivariate levels using simple logistic regression analysis and multiple logistic regression analysis, respectively. Variables selected for inclusion in the multiple logistic regression analysis model were selected using the forward stepwise logistic regression method. Following the fit of the preliminary model, the importance of each variable was verified. The interaction terms were checked using the Likelihood Ratio (LR) test. Multicollinearity problems were was identified by the Variance Inflation Factor (VIF) test.

The final model was assessed for fitness using the Hosmer-Lemeshow goodness-of-fit test. The classification tables for sensitivity and specificity as well as the area under the Receiver Operating Characteristic (ROC) curve were also recorded to assess the model fitness. Influential outliers were identified using Cook's distance. Data points above 1.0 were considered as influential outliers.

## Results

The demographic background of the patients and their satisfaction with their dental appearance are shown in Table 2. Of the 235 patients, (70.2%) were female. Ages ranged from 18 to 62 years with a mean age of 31.5 years (SD 13.0). We found that (52.8%) of these patients were not happy with their general dental appearance, with dissatisfaction with tooth color being the most common (56.2%). In addition, some patients regarded their teeth as poorly aligned (32.3%), crowded (26.4%), and protruding (23.4%). Others reasons for dissatisfaction include self-reported presence of caries (43.4%), non-aesthetic restorations (30.6%), and tooth fractures (15.3%). Patients also answered questions about the treatments they desired to improve their appearance (Table 3). We found that 48.1% wished to have their teeth whitened, followed by restoration of tooth color (18.3%), dentures (16.2%), orthodontic treatment (14.0%), and dental crowns (11.5%).

**Table 2:** Background of patients and satisfaction with dental appearance (n = 235)

Variables	Frequency (%)
Age group (years)	
< 35	146 (62.1)
35 - 54	76 (32.3)
≥ 55	13 (5.5)
	
Sex	
Male	70 (29.8)
Female	165 (70.2)
	
Education	
Primary/Secondary	157 (66.8)
Post secondary/Tertiary	78 (33.2)
	
Satisfied with general dental appearance	
No	124(52.8)
Yes	111 (47.2)
	
Dissatisfied with tooth color	
No	103 (43.8)
Yes	132 (56.2)
	
Felt that teeth were crowded	
No	173 (73.6)
Yes	62 (26.4)
	
Felt that teeth were poorly aligned	
No	159 (66.7)
Yes	76 (32.3)
	
Felt that teeth protruded	
No	180 (76.6)
Yes	55 (23.4)
	
Perceived dental caries	
No	133 (56.6)
Yes	102 (43.4)
	
Perceived non-aesthetic restorations	
No	163 (69.4)
Yes	72 (30.6)
	
Perceived fractures	
No	199 (84.7)
Yes	36 (15.3)

**Table 3:** Desired aesthetic dental treatments (n = 235)

Variables	Frequency (%)
Orthodontic treatment	
No	202 (86.0)
Yes	33 (14.0)
	
Tooth whitening	
No	122 (51.9)
Yes	113 (48.1)
	
Dental crown	
No	208 (88.5)
Yes	27 (11.5)
	
Tooth color restoration	
No	192 (81.7)
Yes	43 (18.3)
	
Denture	
No	197 (83.8)
Yes	38 (16.2)

Table 4 shows a comparison between the 111 patients who were and the 124 who were not satisfied with their general dental appearance. We found that satisfaction with dental appearance differed significantly between males and females. In addition, dissatisfaction with tooth color and perception that of having protruding teeth had significant negative impacts on patient satisfaction with general dental appearance. No other dental problem or condition was associated with patient satisfaction with general dental appearance.

**Table 4:** Profile of patients who were (n = 111) and were not (n = 124) satisfied with their general dental appearance

	Satisfied		χ^2 ^statistics (df)	*p *value
Variables				
	Yes (%) n = 111	No (%) n = 124		
Age group (years)				
< 35	68 (61.3)	78 (62.9)	1.135 (2)	0.567
35 - 54	35 (31.5)	41 (33.1)		
≥ 55	8 (7.2)	5 (4.0)		
				
Sex				
Male	45 (40.5)	25 (20.2)	11.631 (1)	0.001
Female	66 (59.5)	99 (79.8)		
				
Education				
Primary/Secondary	70 (63.1)	87 (70.2)	1 331 (1)	0.249
Post secondary/Tertiary	41 (36.9)	37 (29.8)		
				
Dissatisfied with tooth color				
No	66 (59.5)	37 (29.8)	20.873 (0)	< 0.001
Yes	45 (40.5)	87 (70.2)		
				
Felt that teeth were crowded				
No	84 (75.7)	89 (71.8)	0.459 (1)	0.498
Yes	27 (24.3)	35 (28.2)		
				
Felt that teeth were poorly aligned				
No	80 (72.1)	79 (63.7)	1.872 (1)	0.171
Yes	31 (27.9)	45 (36.3)		
				
Felt that teeth protruded				
No	97 (87.4)	83 (66.9)	13.666 (1)	< 0.001
Yes	14 (12.6)	41 (33.1)		
				
Perceived dental caries				
No	63 (56.8)	70 (56.6)	0.002 (1)	0.962
Yes	48 (43.2)	54 (43.5)		
				
Perceived non-aesthetic restorations				
No	78 (70.3)	85 (68.5)	0.082 (1)	0.775
Yes	33 (29.7)	39 (31.5)		
				
Perceived fractures				
No	94 (84.7)	105 (84.7)	0.000 (1)	0.999
Yes	17 (15.3)	19 (15.3)		

Simple logistic regression analysis of factors influencing patient satisfaction with dental appearance found no significant associations between patient satisfaction and age, education level, perception of having crowded and poorly aligned teeth, self-reported dental caries, non-aesthetic restorations, and fractures of the anterior teeth (Table 5). However, dissatisfaction with general dental appearance was significantly associated with female gender (OR = 2.70, 95% CI: 1.51-4.82), with unhappiness with tooth color (OR = 3.35, 95% CI: 2.01-5.92) and with regarding their teeth as protruding (OR = 3.42, 95% CI: 1.75-6.71).

**Table 5:** Factors influencing patients' satisfaction with dental appearance by simple logistic regression analysis

Variables	Crude OR	95% CI	LR χ^2 ^(df)^a^	*p* value^a^
Age group (years)				
< 35	1.00	-	1.139 (2)	0.566
35 - 54	1.02	0.59-1.78	0.005 (1)^b^	0.941
≥ 55	0.55	0.17-1.75	1.046 (1)^b^	0.306
				
Sex				
Male	1.00	-	11.720 (1)	0.001
Female	2.70	1.51-4.82		
				
Education				
Primary/Secondary	1.00	-	1.330 (1)	0.249
Post secondary/Tertiary	0.73	0.42-1.25		
				
Dissatisfied with tooth color				
No	1.00	-	21.156 (1)	< 0.001
Yes	3.35	2.01-5.92		
				
Felt that teeth were crowded				
No	1.00	-	0.460 (1)	0.498
Yes	1.22	0.68-2.19		
				
Felt that teeth were poorly aligned				
No	1.00	-	1.881 (1)	0.170
Yes	1.47	0.85-2.56		
				
Felt that teeth protruded				
No	1.00	-	14.217 (1)	< 0.001
Yes	3.42	1.75-6.71		
				
Perceived dental caries				
No	1.00	-	0.002 (1)	0.962
Yes	1.01	0.60-1.70		
				
Perceived non-aesthetic restorations				
No	1.00	-	0.082 (1)	0.775
Yes	1.08	0.62-1.89		
				
Perceived fractures				
No	1.00	-	0.000 (1)	0.999
Yes	1.00	0.49-2.04		

^a ^Likelihood Ratio (LR) test

^b ^Wald test

Multivariable logistic regression analysis showed that female gender (OR = 2.18, 95% CI: 1.18-4.03), unhappiness with tooth color (OR = 3.05, 95% CI: 1.74-5.34) and the opinion who felt that their teeth were protruded (OR = 2.91, 95% CI: 1.44-5.91) were significant independent determinants of patient satisfaction with general appearance (Table 6). Possible two-way interactions between factors were not significant, and there was no multicollinearity problem. The preliminary final model was checked for fitness. The result of Hosmer-Lemeshow goodness-of-fit test was not significant (*p *= 0.631, df = 4) and the area under the ROC curve was 0.714, suggesting that the model was fit. The sensitivity and specificity of this model were 64.9% and 64.5% respectively. These results indicated that satisfaction with general dental appearance could be predicted correctly in 64.7% of these patients. When we assessed the contribution of each outlier, we found that none was influential.

**Table 6:** Factors influencing patients' satisfaction with dental appearance by multiple logistic regression analysis

Variables	Adjusted OR	95% CI	LR χ^2 ^(df)^a^	*p* value^a^
Sex				
Male	1.00	-	6.287 (1)	0.012
Female	2.18	1.18-4.03		
				
Satisfied with tooth color				
No	1.00	-	15.568 (1)	< 0.001
Yes	3.05	1.74-5.34		
				
Felt that teeth protruded				
No	1.00	-	9.432 (1)	0.002
Yes	2.91	1.44-5.91		

^a ^Likelihood Ratio (LR) test

Further analyses were performed to evaluate the perceptions of different groups of patients about the color of their teeth. Table 7 shows the distribution of responses by the socio-demographic background (age, sex and education level) between the103 patients who were and the 132 patients who were not satisfied with their tooth color. Results of the chi-square test showed that satisfaction with tooth color differed significantly between male and female patients whereas none of the other background variables was significant. Both simple and multiple logistic regression analyses showed that dissatisfaction with tooth color was significantly higher in female than in males (OR = 1.99; 95% CI: 1.13-3.50).

**Table 7:** Socio-demographic background of patients who were (n = 103) and were not (n = 132) satisfied with their tooth colour

Variables	Satisfied		χ^2 ^statistics (df)	p value
			
	Yes (%) n = 103	No (%) n = 132		
Age group (years)				
< 35	56 (54.4)	90 (68.2)	4.698 (2)	0.095
35 - 54	40 (38.8)	36 (27.3)		
≥ 55	7 (6.8)	6 (4.5)		
				
Sex				
Male	39 (37.9)	31 (23.5)	5.720 (1)	0.017
Female	64 (62.1)	101 (76.5)		
				
Education				
Primary/Secondary	66 (64.1)	91 (68.9)	0.617 (1)	0.432
Post secondary/Tertiary	37 (35.9)	41 (31.1)		

## Discussion

Attitudes and perceptions towards dental appearance differ among populations and among individuals in a population [20]. We found that of adults attending the HUSM dental clinic, only 47.2% were satisfied with the appearance of their teeth, a lower percentage than in previous studies of different populations. For example, a study of 1,014 patients at a dental school in Ankara, Turkey found that (57.3%) were satisfied with their dental appearance [7] as were 76% of stratified sample of adults in the United Kingdom [21].

Perception towards dental appearance is determined by cultural factors and individual preferences varying between individuals and cultures and changing over time [1]. In general, older people (age 55 and above) were more likely than younger people to be satisfied with their dental appearance [7,21], suggesting that the appearance of their teeth is not as important to older than to younger individuals [20]. In this study, however, we found that age was not associated with satisfaction with dental appearance suggesting that dental appearance is becoming equally important in both older and younger adults. This is likely due to the strong impact of the media which portray men and women of all ages as needing to look younger and more beautiful. Indeed, a study of 180 people of six different age strata ranging from 13 to 64 years showed that personal satisfaction with tooth color was age-independent [22]. A study in Sweden of two large samples of 8,881 aged 50 years and 8,563 aged 60 years revealed that the majority of respondents in both groups agreed that beautiful and perfect teeth are very important [23]. Another study on elderly aged 73 to 75 year old in Germany also showed that the importance of dental appearance to overall appearance was rated high by the subjects [24].

Tooth color is a critical factor influencing satisfaction with smile appearance [1]. For example a study in the United Kingdom found that the general public were dissatisfied with relatively mildly discoloured teeth indicating their concern about the color of their teeth [4]. Perception of tooth color is a complex phenomenon that is influenced by many factors including lighting conditions, the optical properties of teeth (translucency, opacity, scattering of light, surface gloss), and the viewer's visual experience [25]. We found that most respondents (56.2%) were dissatisfied with the color of their teeth in agreement with studies in populations in other countries [3,5,7]. In agreement with previous results, we found that, dissatisfaction with tooth color may be the primary reason for dissatisfaction with dental appearance [3].

The important contribution of tooth color to patients' satisfaction with dental appearance was further highlighted by our finding that tooth whitening was the aesthetic treatment most desired by participants, a finding similar to previous results [3]. In addition a study of 180 female patients in South London [6] showed that whitened teeth were preferred over teeth with original color with the former associated with greater attractiveness. In contrast another study in Germany done by Höfel *et al. *[26] found that perceptions of facial attractiveness were independent of tooth color indicating that satisfaction with dental appearance may not correlate positively with facial attractiveness. This finding underlines the influence of psychosocial attributes on the perception of attractiveness.

Many of the patients in this study reported having dental caries and non-aesthetic restorations in their front teeth, with and some reported having tooth fractures. All of these conditions will undoubtedly affect the appearance of teeth, presumably leading to patient dissatisfaction with general dental appearance. Although our patients in this study were not significantly affected by any of those conditions a previous study [3] reported that patient satisfaction with dental appearance was significantly influenced by self-reported caries in anterior teeth, but not by other conditions. Further, decayed anterior teeth were shown to have negative impact on perceptions of facial attractiveness [6].

Patients with high levels of education were found to be more satisfied with the color of their teeth than individuals with lower academic achievement [5,7] as well as to have a lower preference for white teeth [20]. These findings suggested that the higher self-satisfaction with tooth colour observed in individuals with higher academic achievement may reflect higher self-esteem [5,7]. Among our patients the education level did not have impact on satisfaction with tooth color or general dental appearance.

It is a commonly thought that women are more interested in their appearance than men. Indeed, female patients were found to be more concerned with their dental appearance than males [20] as well as to be more critical in judging their dental appearance [24]. Similar to previous results [3] we found that women expressed greater dissatisfaction with dental appearance and tooth color than men. In contrast study in Sweden found that men regarded dental appearance as more important than women [23] while other studies found that the differences were not significant [5,7]. Gender associated differences in satisfaction with dental appearance may require further investigations.

Increased labio-lingual inclination of the anterior teeth may have caused some patients to regard that their teeth as protruding, another factor that influenced patient satisfaction with general dental appearance. We found that, other tooth malalignments did not affect patient satisfaction with general appearance, although self-reported poorly aligned teeth and upper anterior crowding have been found to be associated with patients satisfaction [3,27]. These discrepancies highlighted the wide individual variation in appreciation of acceptable occlusal features. Individuals who perceived their profiles as being different from average were found more likely to be dissatisfied with their facial appearance [28]. Poor tooth alignment and crowding are among the most common malocclusion traits reported in the literature [29-31], which may explain our finding of a lack of association between patients' perceptions of having these traits and satisfaction with general dental appearance.

This study was based entirely on self-reports by patients through an interviewer guided questionnaire. We did not attempt to correlate patient self assessments of their dental problems and their dental records or to compare patients' desired aesthetic treatments and professional assessments of their needs. Furthermore, since the subjects of this study were patients who came to the dental clinic for treatment, they would be expected to be more aware and sensitive to their dental appearance.

## Conclusions

Most patients in this study expressed dissatisfaction with their dental appearance. Dissatisfaction was more common in females than in males. Unhappiness with tooth color and feelings of having protruding teeth also had significant negative influences on patient satisfaction with their general dental appearance. The importance of tooth color was further supported by our finding that most patients would like to have their teeth whitened. These results provide useful indications of the potential demands for dental treatment, particularly aesthetic treatment. In addition, understanding patients' perceptions of their dental appearance is an important aspect of patient management which may assist dentists in planning treatments that are acceptable to the patients leading to higher levels of patient satisfaction.

## Competing interests

The authors declare that they have no competing interests.

## Authors' contributions

MMTO contributed to the design of the study, analyzed the data and wrote the manuscript. NKS contributed to data analyses and interpretation, and revised the manuscript. NH contributed to data acquisition and data management. All authors read and approved the final manuscript.

## Pre-publication history

The pre-publication history for this paper can be accessed here:

http://www.biomedcentral.com/1472-6831/11/6/prepub
